# The role of proteomics in progressing insights into plant secondary metabolism

**DOI:** 10.3389/fpls.2015.00504

**Published:** 2015-07-07

**Authors:** María J. Martínez-Esteso, Ascensión Martínez-Márquez, Susana Sellés-Marchart, Jaime A. Morante-Carriel, Roque Bru-Martínez

**Affiliations:** ^1^Plant Proteomics and Functional Genomics Group, Department of Agrochemistry and Biochemistry, Multidisciplinary Institute for Environmental Studies “Ramon Margalef”, University of Alicante, Alicante, Spain; ^2^Biotechnology and Molecular Biology Group, Quevedo State Technical University, Quevedo, Ecuador; ^3^Proteomics and Genomics Division, Research Technical Facility, University of Alicante, Alicante, Spain

**Keywords:** secondary metabolism, polyphenols, alkaloids, terpenoids, cell cultures, shotgun proteomics, DIGE, MRM

## Abstract

The development of omics has enabled the genome-wide exploration of all kinds of biological processes at the molecular level. Almost every field of plant biology has been analyzed at the genomic, transcriptomic and proteomic level. Here we focus on the particular contribution that proteomic technologies have made in progressing knowledge and characterising plant secondary metabolism (SM) pathways since early expectations were created 15 years ago. We analyzed how three major issues in the proteomic analysis of plant SM have been implemented in various research studies. These issues are: (i) the selection of a suitable plant material rich in secondary metabolites of interest, such as specialized tissues and organs, and *in vitro* cell cultures; (ii) the proteomic strategy to access target proteins, either a comprehensive or a differential analysis; (iii) the proteomic approach, represented by the hypothesis-free discovery proteomics and the hypothesis-driven targeted proteomics. We also examine to what extent the most-advanced technologies have been incorporated into proteomic research in plant SM and highlight some cutting edge techniques that would strongly benefit the progress made in this field.

## Introduction

The development of omics has enabled the genome-wide exploration of all kinds of biological processes at the molecular level. In the field of plant biology, growth and development, organogenesis, stress response or fundamental processes, such as organelle biogenesis, cell cycle or metabolism, have been analyzed at the genomic and proteomic levels (Jorrín et al., 2007; Yuan et al., 2008; Fukushima et al., 2009; Jorrín-Novo et al., 2009, 2015; Gapper et al., 2014). Here we focus on the particular contribution of proteomic technologies to the progress made in the knowledge and characterization of plant secondary metabolism (SM) pathways since the early expectation created 15 years ago (Jacobs et al., 2000). Major interest in this research relies on the discovery of the genes and enzymes involved in the biosynthesis of bioactive and high-value natural products to bridge in the still many knowledge gaps in their respective pathways. On top of all this, the discovery and characterization of the transcription factors involved in the regulation of SM pathways is the ultimate frontier in this field (Yang et al., 2012). Movement of metabolites within cell compartments, between cell types, and even between plant organs, confers a three-dimensional character to the metabolic map. Thus the proteins that act as transporters and carriers have to be included in the pathways. A direct application of such knowledge consists in developing or improving metabolic engineering strategies in different host organisms, including whole plants or plant cells, or microbial systems that are especially suitable for producing the desired metabolites under the cell factory concept (Verpoorte et al., 2000; Yang et al., 2014; Farré et al., 2015). Perhaps one major hurdle for scientists to approach this problem through proteomics is the generally lower abundance of SM proteins, including enzymes, transporters, and especially transcription factors. Other reasons also include: (i) some pathways occur in specialized cells, tissues and organs (Kutchan, 2005), such as the monoterpene biosynthetic pathway in glandular trichomes of peppermint (Turner and Croteau, 2004), which may be difficult to collect in abundance; (ii) fragmented pathways also exist, and each fragment occurs in a different cell type, which can be quite distant within the plant, and some steps can even occur during transport (Kutchan, 2005); e.g., five key enzymes of alkaloid formation in opium poppy occur in multiple cell types and different plant organs (Weid et al., 2004); (iii) each cell or tissue usually contains normal levels of primary metabolism enzymes and housekeeping proteins that involve a high background “noise” of non-target proteins; (iv) some pathways or key enzymes are inducible and are present at a significantly lower level than other enzymes of the same or pathways; (v) most pathways of interest are specific of the under-represented species in sequence databases. Thus model-plant genomic and proteomic information may not be useful as they lack these pathways, at least in part, which precludes extensive protein identification. These issues have been taken into account in some of the studies carried out, but not in them all, and should always be borne in mind for future studies, as well as the limitation of proteomic techniques, which are common to the analysis of samples of any biological origin.

## Plant Material and Secondary Metabolism

**FIGURE 1:**
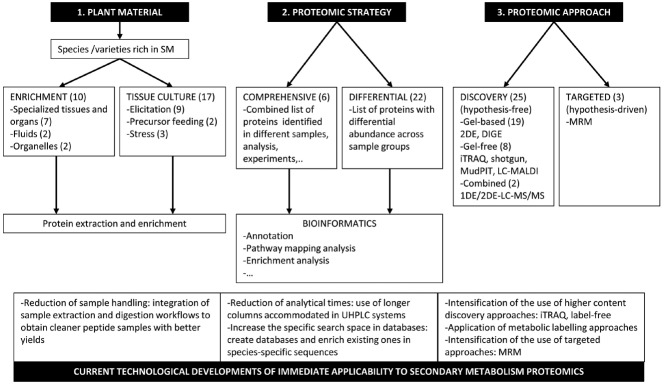
**Major issues in the proteomic analysis of plant secondary metabolism.** Three major issues have been considered in the proteomic analysis of plant secondary metabolism. Two are common to any type of proteomic analysis, i.e., the strategy to find proteins of interest, and the technical approach to achieve it. In the scheme above, we have included the ways in which such issues have been resolved to date and the corresponding number of representative studies (figure in brackets). So the *first and specific issue* for accessing the plant secondary metabolism proteome is the selection of suitable plant material, which is rich in secondary metabolites of interest. If using whole plants as a source, collection of specialised tissues-roots, fruit exocarp and mesocarp-, organs –trichomes-, fluids –milky sap- or preparation of organelles –chromoplasts- before starting protein extraction has been a successful strategy to access the target proteome. Alternatively, *in vitro* cell culture has been a smart option to easily generate an abundant population of homogeneous cells that produce SM whenever they were stimulated through different treatments, such as elicitation, precursor feeding or physical stress. A *second issue* is the proteomic strategy to find target proteins; i.e., enzymes and transporters specifically involved in the metabolic pathway of interest. One is a comprehensive analysis in which the identification of the largest possible number of proteins is intended. The other typical strategy is differential proteomics. In this case, the proteome complements obtained from two experimental groups or more, which differ in secondary metabolite content, are compared. Proteins with differential abundance are selected. In both cases, a bioinformatics-based analysis of the protein lists follows to classify proteins according to their molecular and (potential) biological function, and to select the candidate proteins involved in SM for further functional characterization using biochemical and genetic tools. Eventually, a *third major issue* is the proteomic approach. As the initial goal is to find the new enzymes and transporters involved in secondary metabolite synthesis and biology, a hypothesis-free type discovery proteomics approach, either top-down or bottom-up, is usually undertaken. A number of applications of classical and advanced gel-based and gel-free proteomic techniques to investigate plant SM pathways have been reported. Having identified the proteins of interest, a hypothesis-driven targeted proteomics approach is the next step to profoundly characterize the pathway under different experimental conditions. For this purpose, proteomic workflows have utilized MRM. Indeed a number of technological developments of immediate applicability that are currently used in proteomics from which SM proteomic research would very much benefit are suggested. These may introduce advantages in handling plant material to obtain cleaner and higher yield protein or peptide samples, and to provide improvements in analytical times, protein identification rates, and quantification of protein changes at either the whole or targeted proteome level.

One first issue is to select suitable plant material that is rich in secondary metabolites of interest (Figure 1). In a literature survey about proteomics-based research into plant SM, the first impression is that major families, i.e., phenolics, alkaloids, and terpenes, have been investigated to some extent. However, the high diversity of the metabolic pathways within them is quite under-represented, thus it is necessary to broaden the proteomic research scope in this field. The success of a proteomic approach in finding and discovering new potential pathway enzymes very much relies on the choice of a suitable plant part where the target pathway is over-represented. In grape berry skin, a flesh proteomic analysis has provided extensive coverage of not only shikimate, phenylpropanoid and flavonoid pathway enzymes, but also of their relative abundance profiles during berry development (Martínez-Esteso et al., 2011a,b, 2013). Isolation and comprehensive analyses of trichomes in tobacco (Van Cutsem et al., 2011) and tomato (Schilmiller et al., 2010) have led to the identification of the enzymes involved in the methylerythritol phosphate (MEP) synthesis pathway of terpenoid precursors, terpenoid synthesis and modification, and also potential transporters. The study of tomato, which was combined with a transcriptomic analysis, has also revealed pathways of flavonoid and volatile aldehydes synthesis which occur in trichomes; most interestingly, morphologically identical trichomes from different plant parts appeared to be specialized in the terpenoid metabolite type produced by a sesquiterpene synthase, only found at the protein level in leaf, but not stem, trichomes (Schilmiller et al., 2010). Parts of complex biosynthetic pathways, such as that for alkaloids, may occur in conducting fluids (e.g., phloem and latex). An analysis of latex has revealed the presence of enzymes of SM. Yet a major factor for their successful identification is the availability of the extensive structural annotation of sequences in the databases of the target species. For *Euphorbia kansui* (Zhao et al., 2014), only four of the 19 identified proteins had a functional description, while several hundreds of proteins, including various morphine synthesis steps, have been identified and described for opium poppy (Onoyovwe et al., 2013). The preparation of subcellular fractions specialized in secondary metabolite synthesis, such as chromoplasts from orange fruit pulp (Zeng et al., 2011), has allowed the identification of most of the enzymes of the MEP pathway and lycopene synthesis, and also one enzyme involved in vitamin E. However, it has been noted that it was not possible to identify the enzymes that catalyze the regulated steps in each pathway.

Besides organ and tissues specialized in particular SM pathways, *in vitro* cell cultures have been considered the ideal biological material for equivalence with specialized tissues in which a metabolic pathway occurs or can be induced through elicitation or stress under laboratory controlled conditions. In fact most proteomic studies about SM have been carried out with elicited cell cultures. Among polyphenolics biosynthesis, stilbenoid in grapevine (Martínez-Esteso et al., 2011c; Ferri et al., 2014), flavonolignan in *Silybum marianum* (Corchete and Bru, 2013), lignans in *Podophyllum hexandrum* (Bhattacharyya et al., 2012), isoflavones in *Medicago truncatula* (Lei et al., 2010), and chalcone derivatives in *Boesenbergia rotunda* (Tan et al., 2012) have been analyzed at proteome level under the induction of elicitors, such as chitosan, cyclodextrins, methyl jasmonate or yeast extract, and either individually or combined; i.e., cyclodextrin and methyl jasmonate. In addition to the expected enzymes of the biosynthetic pathway, the bonus proteins, which are potentially involved in movement or modification of end products, have been found to be co-induced; e.g., secretory peroxidases (Martínez-Esteso et al., 2009), glutathione-S transferase (Martínez-Esteso et al., 2011c), Rab11C and ABC transporter (Corchete and Bru, 2013) and laccase (Lei et al., 2010). Alkaloid synthesis has been investigated at the proteome level in cell cultures of California poppy: benzophenanthridine-type (Oldham et al., 2010), opium poppy: benzylisoquinoline-type (Desgagné-Penix et al., 2010), and Madagascar periwinkle: terpenoid indole-type (Champagne et al., 2012), and elicitation and phenotype comparison strategies have been adopted for differential analyses. As these pathways are large and complex, the metabolic profile of cell suspensions may differ from that of plant tissues, even under elicitation. Thus the cell culture approach is useful for analyzing specific parts or branches of the target pathway; e.g., secoiridoid as part of terpenoid indole alkaloid (TIA) synthesis (Champagne et al., 2012), or the sanguinarine branch of benzylisoquinoline alkaloids (BIAs; Desgagné-Penix et al., 2010).

## Proteomic Strategies and Approaches in Secondary Metabolism

A relevant issue that hampers the detection of the specific proteins involved in SM in non-model plant species is the fact that fully sequenced genomes are not available, which represents a significant challenge. Two main approaches have been adopted to overcome the problem in the studies reviewed. Highly conserved proteins can be identified by sequence homology to *Arabidopsis thaliana* and other plant species (Jacobs et al., 2005; Cheng and Yuan, 2006; Bhattacharyya et al., 2012; Nagappan et al., 2012). Alternatively, specific EST databases (Desgagné-Penix et al., 2010; Schilmiller et al., 2010; Champagne et al., 2012) have been created and used for protein identification. Sequence annotation remains a huge challenge as model organisms have only a limited set of SM. The retrieval of Gene Ontology terms from the closest related protein via a BLAST similarity search with the BLAST2GO tool (Conesa et al., 2005) has proven a successful strategy to functionally annotate proteomic experiments in grapevine (Martínez-Esteso et al., 2011a,b) early after the genome sequence release. Although caution is necessary since many annotations are too general, and a minority is manually curated and based on experimental evidence (Yon Rhee et al., 2008).

As depicted in Figure 1, the strategy to find proteins of interest to SM, and the technical approach to achieve this, are in fact common to any proteomic analysis type. Essentially, the strategy consists of a comprehensive analysis, in which the identification of the largest possible number of proteins is intended; or a differential analysis, which focuses only on those proteins with differential abundance across samples. Much of the information obtained in a proteomic comprehensive analysis can be provided through mRNA sequencing at a significantly lower cost and with better dynamic range coverage (Zubarev, 2013), which might be the main reason for it being used much less frequently than differential proteomics as a strategy (Figure 1). Yet the structural and quantitative information obtained through different proteomic strategies still remains an essential tool to identify the acting metabolic enzymes co-localized with the target metabolites in the cell, and to also identify post-translational modifications (PTMs), including proteolytic processing, characterization of protein complexes and, to some extent, the analysis of protein–protein interactions. All of this has given rise to a new way of annotating the genomes called proteogenomics (reviewed in Nevizhskii, 2014). Therefore, the scientific value of undertaking a comprehensive analysis of the global protein expression has been recognized (Baginsky et al., 2010). This strategy has been applied to massively identify and characterize protein content in plant organelles, such as plastids and chromoplast, in specialized plant organs such as trichomes, or in elicited cell cultures.

Since the proteome of a cell or tissue, and even of a purified organelle or cell compartment, is extremely complex, pre-fractionation and protein or peptide separation steps are crucial for broadening SM proteome coverage. The analysis of purified plastid proteomes, followed by differential protein solubilization, has led to the ability to resolve approximately 1,000 different protein species per fraction displayed on 2DE gels (von Zychlinski and Gruissem, 2009). Moreover from purified orange pulp chromoplasts, 418 proteins have been identified as plastid proteins following a shotgun analysis of the protein extract digests (Zeng et al., 2011), of which about 50 were classified as SM biosynthesis. The identification of membrane proteins, especially ABC transporters for their role in SM transport, is particularly important for shedding light on metabolite trafficking across cell and organelle membranes. The shotgun approach is quite suitable for achieving this goal since 2-DE performance for the separation of hydrophobic proteins is compromised. Extensive fractionation and identification of peptides by LC MALDI MS/MS of the microsomal fraction of *Nicotiana tabacum* trichomes, followed by a sequence analysis for transmembrane span prediction, have led to the identification of 165 membrane proteins, of which 39 were putative transporters, and eight were of the ABC type (Van Cutsem et al., 2011). The analysis of whole protein extract digests requires exhaustive separations at the peptide level in order to go more deeply into the proteome. Using multidimensional LC-separation of the peptides mixtures coupled to MS, an increased proteome depth of Madagascar Periwinkle cells has been achieved, compared to conventional 1D-LC peptide separation in shotgun proteomics, and has increased the number of the identified proteins by 40%. Of the 1,663 identified proteins, 22 were involved in TIA biosynthesis and 16 transporters were potentially implicated in SM transport. About 30% of the identified proteins perform an unknown function, which indicates the large gap in SM knowledge. Apart from identification by analogy with other plant species of already characterized proteins, which act in well-defined steps of the TIA biosynthesis pathway, several proteins, including alcohol dehydrogenases, terpene cyclases, cytochrome P450-dependent monooxygenases, glycosyl- and methyl-transferases, have been proposed as candidates to be involved in other unknown steps of this pathway (Champagne et al., 2012). The availability of species-specific nucleotide databases and the technical approach have proved critical to successfully access the SM proteome, which thus demonstrates the power of combining deep mRNA and protein sequencing to access plant-specific SM. Only one enzyme involved in the biosynthesis of BIAs has been identified after a 2-DE-based workflow in opium poppy (*Papaver somniferum*) cell cultures (Zulak et al., 2009a). In contrast, the former creation of a specific EST database from a deep transcriptome analysis, followed by an LC-MS/MS analysis of 1DE-fractionated whole protein extracts, has enabled the identification of 1,004 proteins, which included almost all the enzymes of the pathway (Desgagné-Penix et al., 2010). The creation of a large trichome-specific EST collection and a translated protein database, followed by a similar proteomic workflow on the proteins extracted from mixed and purified type VI tomato trichomes, has led to 1,973 database hits corresponding to 1,552 proteins (Schilmiller et al., 2009, 2010).

In differential proteomics analyses, a significant amount of information can be collected, as reflected in the literature. In the top-down approach, classical 2-DE continues to be the choice for most comparative proteomic studies which aim to find deregulated proteins that act on the metabolic pathways involved in the biosynthesis of important secondary metabolites. A few key enzymes directly related to anthocyanin biosynthesis, and their accumulation on the skin of grape berries, have been detected while studying the effect of a low leaf-to-fruit ratio (Wu et al., 2013), sunlight exclusion (Niu et al., 2013b) and the difference of the red-to-white cultivar (Niu et al., 2013a). In two other studies, no clear results on the successful finding of SM enzymes have been obtained by 2-DE differential proteomics (Nagappan et al., 2012; Zhao et al., 2014). This could be due, in part, to the limitations of 2DE to access a lower abundance protein population when whole protein extracts are being analyzed. Apart from exhaustive fractionation, the use of advanced enrichment strategies to detect low abundant proteins such as combinational hexapeptide ligand libraries (CPLL) combined with 2-DE has been applied to analyze the effect of UV stress on *Mahonia bealei* leaves in relation to the induction of the BIAs alkaloid production. As a result, 91 deregulated proteins have been identified, but only after CPLL enrichment, which has led to the finding of several new SM proteins (Zhang et al., 2014).

In the cell cultures that produce SM in response to stimuli, the extent of SM identified proteins in whole protein extracts has slightly improved compared to plant tissues. For example, several phenylpropanoid and monolignol biosynthetic enzymes accumulated in methyl jasmonate-elicited *Podophyllum hexandrum* cell cultures (Bhattacharyya et al., 2012); isoflavonoid biosynthetic enzymes and a putative laccase have been found to accumulate in yeast-elicited *Medicago truncatula* cell cultures (Lei et al., 2010); soluble and microsomal isoforms of stilbene synthase have been identified in chitosan-elicited *Vitis vinifera* cell cultures (Ferri et al., 2014); up to eleven proteins involved in the phenylpropanoid biosynthetic pathway have been found to be up-regulated upon feeding *B. rotunda* callus with phenylalanine to induce the accumulation of chalcone derivative SMs (Tan et al., 2012).

Differential proteomics methods that better perform than single staining 2-DE have also been applied to conduct discovery studies in plant SM. These include differential in gel electrophoresis (DIGE), as a top-down protein-centric approach (Martínez-Esteso et al., 2011c; Champagne et al., 2012; Corchete and Bru, 2013), and isobaric tags for relative and absolute quantitation (iTRAQ) (Martínez-Esteso et al., 2011b, 2013; Robbins et al., 2013) and label-free (Oldham et al., 2010), as bottom-up peptide-centric approaches. DIGE has allowed the identification of three TIA biosynthetic enzymes in *Catharanthus roseus* cell cultures and five others that are putatively involved in the pathway among the 172 identified deregulated proteins (Champagne et al., 2012). The comparison made of DIGE and iTRAQ results has shown a good quantitative correlation between commonly identified proteins (Robbins et al., 2013), but iTRAQ is clearly superior in proteome coverage. In developing and ripening grape berries, DIGE has identified six flavonoid pathway enzymes present in 15 spots (Martínez-Esteso et al., 2011b), while 38 proteins from the shikimate, phenylpropanoid and flavonoid pathways have been identified with iTRAQ (Martínez-Esteso et al., 2013). However, the observed partial overlap between both types of proteome analyses emphasizes the use of different approaches to gain a better picture of the target process under study.

The question as to what approach to use very much depends on the type of information sought. Gel-free approaches combined with extensive fractionation results in broader proteome coverage as compared to gel-based methods. Gel-based techniques greatly benefit the detection of isoforms and post-translationally modified proteins.

Having identified the proteins involved in a SM pathway of interest, a targeted proteomics approach with a multiple reaction monitoring (MRM) methodology in triple quadrupole instruments can be accomplished to study their quantitative behavior under different experimental conditions or to functionally analyze protein isoforms. And so it was that Norway spruce isoforms of terpene synthases and 1-deoxy-D-xylulose-5-phosphate synthase (DXS) (Zulak et al., 2009b), and loquat polyphenol oxidase isoforms (Martínez-Márquez et al., 2013) have been quantitatively analyzed. Pre-fractionation of peptides can also be implemented to improve signal quality in MRM. In the MRM analysis of strawberry proteins related to flavonoid and anthocyanin biosynthesis during ripening, peptides have been previously fractionated based on pI (OFFGEL; Song et al., 2015).

## Future Prospects

Based on all this information, it is possible to find some important messages: (i) databases need to be enriched in species-specific sequences as this clearly increases the rate of identification of the proteins that act on SM pathways; (ii) gel-based differential analyses are less effective than Gel-free ones to access lower abundance SM proteins, but as the information provided by each technique is only partly redundant, a combined approach is recommended; (iii) plant material selection and sample fractionation are critical for good coverage of SM pathways. Moreover, there are a number of technological developments that are currently used in proteomics of immediate applicability from which SM proteomic research would greatly benefit. Some important ones are described below.

Proteomic approaches and workflows have evolved very quickly in recent years and are ready to be exploited to analyze the plant SM proteome more profoundly; i.e., better resolution chromatography in relatively longer columns accommodated in ultra-HPLC systems, which enables similar performance as extensive 2D-LC fractionation to be achieved in a single shot (Nagaraj et al., 2012).

Metabolic isotope labeling strategies, which are still not used in SM proteomics, are especially suitable for using plant cell cultures fed with isotopic variants of amino acids (Gruhler et al., 2005), known as SILAC, or fed with cost-effective ^15^N-KNO_3_ salt as the sole N source (Engelsberger et al., 2006). The latter is also applicable to whole plants grown in hydroponics (Ippel et al., 2004). Compared to chemical labeling, i.e., iTRAQ, metabolic labeling has the advantage of simplifying sample preparation and handling as heavy and light variants can be mixed at the plant material level to thus reduce technical variability. A major bottleneck of using ^15^N-labeling as a differential proteomics strategy is the variable mass shift between the light and heavy forms, dependent on amino acid composition, which implies adding considerable complexity to the quantitative data analysis. The application of SILAC to plant cell cultures has been shown to be restricted to incomplete isotopic label incorporation because plants are able to “*de novo*” synthesize all proteinogenic amino acids, and to thus compete with the labeled amino acids fed into the culture medium. Recently, a smart modification of the SILAC protocol has enabled the accurate reproducible application of SILAC to plant cells grown in the dark (Schütz et al., 2011), thus providing a new powerful tool to analyze the SM proteome.

The spectacular development of instrumentation for LC-MS of peptides over the last decade has almost left protein sample preparation, including extraction and digestion, as the one major critical point in proteomic workflows in the overall performance of proteomic experiments. Cleanness of samples in relation to non-protein contaminants dramatically affects the protein identification rate. The current trend in simplifying sample preparation steps and handling minimal quantities of biological material has led to the integration of protein extraction, digestion, and fractionation in a single pipette tip that holds a small disk of membrane-embedded separation material, the so-called StageTip (Rappsilber et al., 2003; Kulak et al., 2014). Extrapolating these protocols to plant material is challenging given protein scarcity and the abundance of interfering compounds in plant cells, but it is an exciting challenge because the benefits for research of SM will outweigh development efforts.

Targeted proteomics using MRM is a powerful proteomic approach that has been scarcely used to investigate plant proteomics, and more specifically SM proteomics. This hypothesis-driven relatively new approach requires the previous selection of the target proteins and their proteotypic peptides to monitor and/or quantify them either in a relative or an absolute fashion (Lange et al., 2008). Since the availability of genome-wide sequences, as well as annotated spectral libraries, are critical in the application of this methodology, plants are currently at a disadvantage compared to mammalian and microbial organisms. Future efforts in plant proteomics should generally focus on this issue. In addition to the above-cited examples (Zulak et al., 2009b; Martínez-Márquez et al., 2013; Song et al., 2015), MRM might become the gold standard to validate proteomic experiments as an alternative to using western blotting (Lehmann et al., 2008; Aebersold et al., 2013), and it could be particularly relevant in plant SM proteomics thanks to the highly limited availability of specific antibodies. Quantification of target enzymes allows a much larger number of conditions analyzed per experiment, which accelerates the process to reveal key enzymes. As the number of unveiled target proteins, i.e., enzymes and transporters, directly involved in the SM pathways increases, it is envisaged that this approach will be used extensively in the near future.

### Conflict of Interest Statement

The authors declare that the research was conducted in the absence of any commercial or financial relationships that could be construed as a potential conflict of interest.
